# Sleep loss effects on physiological and cognitive responses to systemic environmental hypoxia

**DOI:** 10.3389/fphys.2022.1046166

**Published:** 2022-12-12

**Authors:** Pierre Fabries, Danielle Gomez-Merino, Fabien Sauvet, Alexandra Malgoyre, Nathalie Koulmann, Mounir Chennaoui

**Affiliations:** ^1^ REF-Aero Department, French Armed Forces Biomedical Research Institute—IRBA, Brétigny-sur-Orge, France; ^2^ Laboratoire de Biologie de l’Exercice pour la Performance et la Santé (LBEPS), UMR, Université Paris-Saclay, IRBA, Evry-Courcouronnes, France; ^3^ French Military Health Academy—Ecole du Val-de-Grâce, Place Alphonse Laveran, Paris, France; ^4^ Vigilance Fatigue Sommeil et Santé Publique (VIFASOM) URP 7330, Université de Paris Cité, Paris, France

**Keywords:** sleep, altitude, physiology, acute mountain sickness, cognition

## Abstract

In the course of their missions or training, alpinists, but also mountain combat forces and mountain security services, professional miners, aircrew, aircraft and glider pilots and helicopter crews are regularly exposed to altitude without oxygen supplementation. At altitude, humans are exposed to systemic environmental hypoxia induced by the decrease in barometric pressure (<1,013 hPa) which decreases the inspired partial pressure of oxygen (PIO_2_), while the oxygen fraction is constant (equal to approximately 20.9%). Effects of altitude on humans occur gradually and depend on the duration of exposure and the altitude level. From 1,500 m altitude (response threshold), several adaptive responses offset the effects of hypoxia, involving the respiratory and the cardiovascular systems, and the oxygen transport capacity of the blood. Fatigue and cognitive and sensory disorders are usually observed from 2,500 m (threshold of prolonged hypoxia). Above 3,500 m (the threshold for disorders), the effects are not completely compensated and maladaptive responses occur and individuals develop altitude headache or acute altitude illness [Acute Mountain Sickness (AMS)]. The magnitude of effects varies considerably between different physiological systems and exhibits significant inter-individual variability. In addition to comorbidities, the factors of vulnerability are still little known. They can be constitutive (genetic) or circumstantial (sleep deprivation, fatigue, speed of ascent.). In particular, sleep loss, a condition that is often encountered in real-life settings, could have an impact on the physiological and cognitive responses to hypoxia. In this review, we report the current state of knowledge on the impact of sleep loss on responses to environmental hypoxia in humans, with the aim of identifying possible consequences for AMS risk and cognition, as well as the value of behavioral and non-pharmacological countermeasures.

## 1 Introduction

Climbing to altitude involves a non-linear decrease in barometric pressure, which leads to a decrease in inspired oxygen partial pressure (PIO_2_) and, subsequently, a decrease in oxygen partial pressure (PO_2_) at each point in the oxygen transport cascade (alveolar, arterial, tissue and venous blood) (Hurtado, 1964). The effects of altitude occur gradually and depends of the duration of exposure and altitude level. The reduction in PO_2_ results in several physiological responses over a period of minutes to weeks after the initial exposure (Luks, 1985; Marotte, 2004; Bärtsch and Gibbs, 2007; Burtscher et al., 2019). These responses are intended to promote acclimatization of the individual to compensate for hypoxic conditions. However, most unacclimatized subjects (50%–85%) traveling to an altitude between 4,500 and 5,000 m above sea level will develop acute mountain sickness (AMS) or experience symptoms (Maggiorini et al., 1990; Bärtsch and Swenson, 2013). Maladaptive reactions to high altitude present as one of three forms of acute altitude illness, namely AMS (combining headache with gastrointestinal symptoms, fatigue/weakness, dizziness and/or sleep disturbances, at different levels of severity), high altitude cerebral edema (HACE) or high altitude pulmonary edema (HAPE) (Linde et al., 2017; Luks et al., 2017). Exposure to high altitude also induces cognitive impairments, fatigue, headaches and sleep disorders (e.g., Cheyne-stoke breathing, increased wake after sleep onset and sleep onset latency, decrease in total sleep time) (Beaumont et al., 2007; de Aquino Lemos et al., 2012; Heinzer et al., 2016), sometimes linked to the environmental conditions of trekking (jet lag due to travel, sleeping in high-altitude refuges, departure at night or very early in the morning, cold weather), similar to an isolated, confined and extreme (ICE) environment (Zivi et al., 2020).

More and more lowlanders people are exposed to high altitude either for pleasure (mountaineering) or for work (military, high mountain guides, etc.), and would therefore be concerned by the importance of avoiding sleep deprivation/loss before altitude exposure. Indeed, sleep is considered crucial for the maintenance of physiological and psychological functions. Numerous human studies have shown that sleep deprivation (SD), whether total or partial, induces physiological and psychological disturbances, increases perceived fatigue, impairs cognitive functions (e.g., sustained attention, decision making, executive functions, etc.) and generally deteriorate physical capacity (Killgore, 2010; Arnal et al., 2015; Troxel et al., 2015; Rabat et al., 2016; Grandou et al., 2019). Given the critical role of sleep in regulating physiological systems, it can be hypothesized that sleep debt prior to exposure to high altitude could disrupt physiological responses, and increase the risk of developing AMS. The objective of this brief narrative review is to show that sleep deprivation can influence respiratory, cardiovascular and cognitive responses to environmental hypoxia, in relation to the risk of AMS. Our review does not target sleep pathologies involving sleep loss or fragmentation.

## 2 Physiological and cognitive responses to sleep loss

Sleep is a fundamental physiological need for human life. For most individuals, sleep occupies between 20% and 40% of a 24-h day and provides multiple restorative functions for the body and brain. It affects temperature regulation and many systems such as the autonomic nervous system, the cardiovascular and respiratory systems, the immune system, etc. (Colten and Altevogt, 2006; Besedovsky et al., 2012; Sowho et al., 2014). It regulates endocrine function and synaptic homeostasis, while playing an important role in immune and inflammatory control, metabolism and tissue repair (Copinschi et al., 2014; Eugene and Masiak, 2015; Irwin, 2015; Chennaoui et al., 2020; Lange et al., 2022). Sleep also improves reaction time and memory recall through its role in synaptic homeostasis, and reduces mental fatigue (Copinschi et al., 2014; Eugene and Masiak, 2015; Irwin, 2015; Chennaoui et al., 2020; Lange et al., 2022).

### 2.1 Cardiovascular, inflammatory and metabolic responses

The known forms of sleep loss have been defined as total SD, chronic sleep restriction (also sometimes referred to as partial SD), and sleep fragmentation or disruption (Reynolds and Banks, 2010). Total SD is the suppression of sleep for a given period of time (at least one night) and it has been widely researched in laboratory protocols. Sleep restriction is a reduction in sleep time below an individual’s usual amount, or the regular amount of sleep needed to maintain optimal performance.

SD has been found to lead to several biological consequences, such as autonomic dysregulation, increased oxidative stress, or altered endocrine and inflammatory responses (Mullington et al., 2009; Chennaoui et al., 2011; Irwin et al., 2016; Atrooz and Salim, 2020). Studies on both humans and rodents have shown that SD and sleep restriction are often associated with small and temporary increases in the activity of the major neuroendocrine stress systems, i.e., the autonomic sympatho-adrenal system and the hypothalamic-pituitary adrenal axis (HPA) (Meerlo et al., 2008). Indeed, levels of norepinephrine and epinephrine have been found significantly enhanced after partial SD in the late night (awake from 0300 to 0600 h), and plasma cortisol levels over the 1,800–2,300 h period were 37% and 45% higher on the day following acute partial SD (sleeping from 0400 to 0800 h) and total SD than on the previous day in healthy male volunteers (Leproult et al., 1997; Irwin et al., 1999). However, divergent findings have also been reported and discrepancies could be due to experimental conditions and/or frequency of blood sampling. In addition, epidemiological and experimental studies have confirmed that SD is associated with increased blood pressure (BP) in healthy subjects, and adverse cardiovascular events (Sauvet et al., 2010; Liu and Chen, 2019). Sleep loss is considered as a risk factor for cardiovascular events mediated by endothelial dysfunction, and hormonal and inflammatory responses. Indeed, endothelial dysfunction is currently recognized as a key early factor in the development of atherosclerosis, and is associated with numerous risk factors for cardiovascular disease, including hypertension, coronary artery disease, vascular calcifications, heart failure with preserved ejection fraction, sudden death and the metabolic syndrome (Anderson et al., 1995; Davignon and Ganz, 2004). In laboratory experimental protocols, acute total SD or repeated partial deprivation (also named sleep restriction) cause significant increases in cardiac and peripheral sympathetic modulation, associated with mild and subclinical endothelial dysfunction and increased arterial stiffness in healthy young male subjects (Sauvet et al., 2010; Dettoni et al., 2012; Sunbul et al., 2014; Sauvet et al., 2015). It has been suggested that SD-related endothelial dysfunction is triggered by factors such as sympathetic activation and low-grade inflammatory responses.

Sleep is thought to play an important role in blood sugar control. Recurrent sleep restriction has been shown to have deleterious effects on carbohydrate metabolism and endocrine function (Spiegel et al., 2009). Sleep restriction also increased the levels of inflammatory mediators, contributing to the development of metabolic disorders and cardiovascular diseases. Recurrent sleep restriction induces metabolic and endocrine alterations leading to increased hunger and appetite, and weight gain (e.g., increased ghrelin levels, decreased leptin levels, decreased glucose tolerance, decreased insulin sensitivity, increased evening cortisol concentrations.) (Spiegel et al., 2004; Bayon et al., 2014). In healthy subjects, SD can also cause an increase in white blood cells and other markers of inflammation to levels associated with the future development of cardiovascular diseases (Mullington et al., 2009). Subclinical increases in plasma concentration or expression in blood cells of the proinflammatory cytokines interleukin-6 (IL-6) and tumor necrosis factor alpha (TNF-α), and C-reactive protein (CRP) were found during acute total SD and also during sleep restriction (Meier-Ewert et al., 2004; Chennaoui et al., 2011; Irwin et al., 2016; Sauvet et al., 2017). Cellular inflammatory signaling is activated due to the activation of nuclear factor-Kappa B (NF-κB), the key transcriptional control pathway in the inflammatory signaling cascade (Irwin et al., 2008).

### 2.2 Respiratory responses

The regulation of breathing is ensured by sensors, control centers and effectors (Berger and Nadel, 1988; Braman, 1995). Two categories of sensors control breathing: sensory receptors and chemoreceptors. The latter are sensitive to variations in the concentration of oxygen, carbon dioxide and hydrogen ions in the blood. They send impulses to the control centers (located in the brainstem) which adapt the ventilation by acting on the effector organs (the respiratory muscles) (Braman, 1995).

The influence of SD on respiratory motor output has been poorly investigated. In the basal state for humans under eupneic conditions, it has been repeatedly demonstrated that one night of SD does not alter the functions of the ventilatory system: there is no change in ventilation or gas exchange (Chen and Tang, 1989; Spengler and Shea, 2000; Rault et al., 2020).

In contrast, when changing the gas composition of the inspired air in order to assess adaptive capacities, an altered ventilatory response to hypercapnia and to hypoxaemia was described following one night of SD (Schiffman et al., 1983; White et al., 1983). However, several authors have not reproduced the hypercapnic ventilator response to SD in humans (Leiter et al., 1985; Spengler and Shea, 2000). In Spengler and Shea (2000) demonstrated that SD *per se* does not reduce the sensitivity of central chemoreceptors and have suggested that associated confounding factors may be involved.

However, total SD has been found to decrease skeletal muscle endurance (the diaphragm and intercostal muscles) (Martin, 1981; Roberts et al., 2019), with a contradictory effect on maximal oxygen consumption, which is not or only slightly but significantly decreased (Horne and Pettitt, 1984; Plyley et al., 1987). The mechanisms behind the impairment of endurance performance after sleep loss are still unclear. With respect to effects of SD on the endurance of the ventilatory muscle function, a recent study in healthy subjects with normal diaphragm function has evidenced that one night SD reduces respiratory motor output by altering its cortical component with a subsequent reduction (50%) in inspiratory endurance (Rault et al., 2020). Thus, these authors suggested that impaired sleep triggers severe brain dysfunction that could precipitate respiratory failure. Their subsequent work demonstrated that SD abolished vagal tone response to inspiratory load, possibly contributing to a higher heart rate during the inspiratory loading trial and reduced inspiration endurance (Westphal et al., 2021).

### 2.3 Cognitive responses

Sleep loss is known to impair multiple neurobehavioral functions, such as reaction time for sustained attention and inhibition and working memory for executive functions, including in athletes, and induce daytime sleepiness, despite motivation to prevent these effects (Van Dongen et al., 2003; Reynolds and Banks, 2010; Basner et al., 2013; Vitale et al., 2019). In addition, SD may limit the benefits of sleep for memory consolidation. However, there are large interindividual differences in the degree of decline in cognitive performance over the course of SD, depending on the task and performance metric under consideration (Killgore, 2010; Hudson et al., 2020). There are interindividual, trait-like phenotypic differences related to neurobehavioral deficits during SD, such that some individuals are minimally affected by sleep deprivation (i.e., resilient) and others are highly affected (i.e., vulnerable) (Van Dongen et al., 2004; Goel et al., 2009; Killgore, 2010).

The specific areas of the brain that are vulnerable to SD were studied using functional magnetic resonance imaging (fMRI) and positron emission tomography (PET) (Basner et al., 2013). Early studies using PET revealed a reduction in metabolic rate in the thalamic, parietal, and prefrontal regions during prolonged SD of 24–72 h. Reduced fronto-parietal activation during lapses on a visual selective attention task has been evidenced after one night of acute total SD, using blood oxygenation level dependent fMRI. Sleep-deprived vulnerable individuals showed a reduction in frontoparietal signaling while resilient individuals showed a tendency to increase fronto-parietal activation. In the human brain, PET studies also evidenced a down-regulation of striatal D2/D3 dopamine receptors and an increase of the serotonin receptor binding potential in a variety of cortical regions. Changes in cerebral blood flow (CBF) were also demonstrated after a day of wakefulness, sleep and SD (Basner et al., 2013; Elvsåshagen et al., 2019). Finally, functional connectivity alterations have been evidenced following SD using resting-state functional connectivity (Chee and Zhou, 2019).

It is now known that one of the brain neuromodulators that may be involved in SD-induced changes in cognitive functioning is the adenosine system and its dynamically regulated receptors, which is considered an important sleep-regulating substance (Bjorness and Greene, 2009; Elmenhorst et al., 2017). Adenosine is a metabolic intermediate of adenosine tri-phosphate (ATP). Adenosine can cross the blood-brain barrier and has neuromodulatory properties; it is involved in sleep promotion and suppression of arousal with increased daytime wakefulness. Studies from Elmenhorst et al. (2007) team demonstrated *in vivo* that SD resulted in increased adenosine A1 receptor (A_1_R) availability and high individual A_1_R availability is related to a low sleep pressure and good cognitive performance (Elmenhorst et al., 2017). Further evidence for a role of adenosine in modulating sleep pressure and the associated variation in wakefulness and attention stems from the effects of the adenosine receptor antagonist caffeine that attenuates neurobehavioral consequences of SD (Urry and Landolt, 2015; Erblang et al., 2021a). Finally, several endogenous factors are thought to contribute to the cognitive consequences of severe sleep deficits including the growth hormone/insulin-like growth factor-1 (GH/IGF-1) trophic system, brain-derived neurotrophic factor (BDNF), hormones, and cytokines (Kreutzmann et al., 2015; Chennaoui et al., 2020). There are also genotype-dependent interindividual differences in phenotypic neurobehavioral responses to sleep deprivation or restriction (Bodenmann et al., 2012; Erblang et al., 2021b; Casale and Goel, 2021).

## 3 Physiological and cognitive responses to systemic environmental hypoxia

Exposure to high-altitude environments above 3,000 m for periods ranging from several hours to days presents a challenge to the human body due to the gradual reduction in barometric pressure and subsequent reduction in oxygen pressure, resulting in a series of important physiological responses (respiratory, cardiovascular, cerebrovascular, hematological and metabolic) that enable individuals to tolerate hypoxia and ensure tissue oxygen supply (Luks, 1985; Cogo, 2011). The physiological responses are mediated through chemosensitivity on the carotid bodies (CB) and intracellular signaling by hypoxia-inducible factors (HIFs), whereas others occur in a HIF-independent manner. The absence or excess of these hypoxia-induced physiological responses are responsible for maladaptative responses and primary altitude illnesses, foremost among them AMS (Klocke et al., 1998; Bartsch et al., 2004; Burtscher et al., 2021). However, the prevalence of AMS is highly dependent on the study setting: for Luks et al. (2017) it varies between 40% and 90%, depending on altitude and individual susceptibility, in unacclimatized individuals ascending more than 500 m per day at altitudes of 4,500–6,000 m, whereas it is ∼25%–40% when passively ascending at 3,000–3,500 m. Hypoxia also has detrimental neurobehavioral effects for unacclimatized humans: alterations in cognitive function, mood and sleep quality occur after a high-altitude climb (Bahrke and Shukitt-Hale, 1993; Ainslie et al., 2013; Lombardi et al., 2013; McMorris et al., 2017). Interestingly, it has recently been shown that AMS and sleep disturbances differentially influence cognition and mood during rapid ascent (in a hypobaric chamber) to 3,000 and 4,050 m: the presence of AMS impacted mood while poor sleep impacted cognition (Figueiredo et al., 2022).

### 3.1 Respiratory and cardiovascular responses

Systemic exposure to hypoxia is an important physiological stimulus that induces adaptive (homeostatic) responses: acute oxygen sensing is necessary for the activation of cardiorespiratory reflexes that allow individuals to survive in a hypoxic environment (López-Barneo et al., 2016). CB chemoreceptors, located at the bifurcation of carotid arteries, are sensors of arterial O_2_, CO_2_, and pH (Kumar and Prabhakar, 2012). These peripheral receptors function in cooperation with central chemoreceptors in the brain that monitor CO_2_-pH or O_2_ (Lindsey et al., 2018). Decreased arterial O_2_ levels increase CB sensory nerve activity (Kumar and Prabhakar, 2012). The chemosensory reflex from the CB is a major regulator of breathing (hyperventilation) and sympathetic nerve activity (with increased catecholamines, heart rate and arterial vasoconstriction increasing BP) (Semenza and Prabhakar, 2018).

#### 3.1.1 Detection of acute hypoxia by the carotid bodies (CB)

CB tissue consists of two major cell types: type I cells (glomus cells), which are neuronal in origin, and type II cells (glial cells). The hypoxic sensing in type 1 cell appears to be mediated by the heme oxygenase-2 (HO-2) signaling pathway (Prabhakar et al., 2018). HO-2 is a carbon monoxide (CO)-producing enzyme (Yuan et al., 2015), and NADH dehydrogenase Fe-S protein 2, a subunit of the mitochondrial complex I. The HO-2 seems to appear as a major regulator upstream the hypoxic pathway. O_2_-dependent CO production from HO-2 mediates hypoxic response of the CB by regulating the generation of H2S (Prabhakar et al., 2018). Hypoxia inhibits certain K^+^ channels in type I cells and the resulting depolarization results in Ca^2+^-dependent release of one or more neurotransmitters, which stimulate the neighboring sensory nerve terminal, resulting in increased sensory discharge (Kumar and Prabhakar, 2012).

In the high-altitude adapted Tibetan population characterized by increased resting ventilation, better blood oxygen saturation and reduced hemoglobin (Hb) levels (Wu and Kayser, 2006; Beall, 2007), a genetic variation of *HMOX2* (heme oxygenase-2) has been found associated with different levels expression of HO-2 and consequences on the Hb levels: carriers of the C allele at rs4786504_*HMOX2*:T>C displayed lower Hb level than the T/T homozygous (Yang et al., 2016). In this study, it was also evidenced *in vitro* that the C allele could increase the expression of *HMOX2*. Furthermore, HMOX2 is one of several candidate genes involved in high-altitude adaptations, which include in particular, *EPAS1* (Endothelial PAS domain- contains Protein 1) and *EGLN1* (Egl-9 family hypoxia inducible factor 1) two upstream regulators of the HIF pathway in Tibetans (Lorenzo et al., 2014; Peng et al., 2014; Bigham, 2016).

#### 3.1.2 Central integration and respiratory responses

Afferent signals from the CB travel to the nucleus of the solitary tract (NTS) in the brainstem medulla. From the NTS there are multiple neural projections to the respiratory and autonomic centers resulting in increased ventilatory drive (Pamenter and Powell, 2016; Kane et al., 2020). Normally, the pulmonary response to acute exposure to altitude is primarily hyperventilation to ensure adequate oxygen delivery to the tissues. At rest, ventilation increases by first increasing the tidal volume, at least up to 3,500 m. Above this altitude, there is also the significant increase in breathing rate (Cogo, 2011).

Under isocapnic conditions, where PACO_2_ is experimentally maintained by adding CO_2_ to inspired gas, this response is higher than under poikilocapnic conditions for which PACO_2_ can vary with alveolar ventilation. In both conditions, the initial ventilatory response is followed by a decrease in ventilation, called the hypoxic ventilatory decline, partly related to hypocapnia. If hypoxia is maintained for hours or days (i.e., becomes chronic, e.g., high altitude ascent), there is a further increase in ventilatory drive. It has been classically proposed that urinary loss of HCO_3_^−^ contribute to compensate this ventilatory alkalosis in addition to a central resetting of CO_2_-sensitive chemoreceptors. Changes in central chemosensitivity parameters, such as decreased ventilatory response threshold to hypercapnia, are observed after exposure to hypoxia (Mahamed et al., 2003; Richard and Koehle, 2012).

#### 3.1.3 Cardiovascular and cerebrovascular responses

Acute hypoxia results in cardiovascular responses to maintain oxygen in vital tissues. The response is complex with a centralized neural and endocrine response that results in sympathetic activation with increased sympathetic nerve activity, heart rate, and systolic and diastolic blood pressure in humans (Xie et al., 1985; Ainslie and Poulin, 2004; Richalet, 2016; Mallet et al., 2021). The increase in cardiac output to maintain oxygen delivery to tissues is largely due to the increase in heart rate caused by pronounced sympathetic activation. The latter also induced an increase in systemic BP during acute exposure to altitude. Previous studies have indicated that cardiorespiratory performance (as assessed by maximal oxygen consumption, VO_2_max) decreases by approximately 1.5%–3.5% for each additional 300 m of elevation above 1,500 m (Burtscher et al., 2015). This decline is more pronounced at altitudes above 5,000 m (Latshang et al., 2013).

Hypoxia-induced physiological vasoconstriction in the pulmonary circulation is also responsible for increased pulmonary artery pressure and endothelial permeability, which may explain the extravascular accumulation of pulmonary fluid described in a few articles (Cremona et al., 2002; Cogo, 2011). Indeed, high-altitude exposure has been evidenced to decrease nitric oxide (NO) availability (Brown et al., 2006) and induce a systemic inflammatory response and endothelial activation/injury as shown by increases in endothelin-1 (ET-1) and the inflammatory IL-6 cytokine (Boos et al., 2016a). In addition, with increasing altitude, flow-mediated dilation (FMD) decreases, resulting in increased vascular permeability and decreased antioxidant capacity (Boos et al., 2012). Moreover high-altitude natives presented lower plasma ET-1 levels compared to lowlanders, and this is indicative adaptation to hypoxic stress (Rajput et al., 2006).

Exposure to hypoxia produces a marked increase in CBF, proportional to the severity of hypoxia, followed by a gradual decrease towards normal values. It depends on many physiological factors and varies considerably depending on the nature and duration of exposure (Ainslie and Subudhi, 2014). The magnitude of CBF change at high altitude is influenced by many variables, including PaO_2_ and PaCO_2,_ oxygen content, cerebrospinal fluid pH, and hematocrit, but can be collectively summarized in terms of the relative strengths of four key built-in reflexes, i.e., hypoxic cerebral vasodilatation, hypocapnic cerebral vasoconstriction, hypoxic and hypercapnic ventilatory responses. In addition to these reflex responses, which are most likely adjusted during the acclimatization process, the CBF is also influenced by a myriad of other hypoxia-induced changes including adenosine, angiogenesis, nitric oxide, blood viscosity/hematocrit, HIF, endothelial growth factor (VEGF), and free radicals (Ainslie and Subudhi, 2014; Hoiland et al., 2018).

#### 3.1.4 Respiratory, cardiovascular and cerebrovascular responses and AMS

Several factors for susceptibility to AMS have been identified including a history of previous AMS, the speed of ascent (Hsu et al., 2015; Poudel et al., 2022), baseline SpO_2_ (Guo et al., 2014; Boos et al., 2016b), age, sex (Hou et al., 2019), smoking (Vinnikov et al., 2016a), obesity (Ri-Li et al., 2003), the studied population (e.g., mine workers) (Vinnikov et al., 2014), and individual susceptibility (Schneider et al., 2002).

Among the physiological factors for AMS, it has been hypothesized that people with a blunted hypoxic ventilatory response (HVR) would be more likely to suffer from AMS than those with a fast HVR, such as AMS-resistant people. Chemosensitivity parameters (high hypoxia desaturation and low HVR at submaximal exercise) were shown to be independent predictors of AMS in a large population undergoing outpatient altitude medicine consultation (Richalet et al., 2012). Thus, AMS-susceptible subjects have a lower HVR compared with their AMS-resistant counterparts (Moore et al., 1986; Nespoulet et al., 2012).

Increased sympathoadrenal activity has been implicated in the pathogenesis of AMS; during an 8-h exposure to normobaric hypoxia (12% O_2_, equal to 4,600 m) subjects who developed AMS had significantly higher arterial epinephrine concentrations (Kamimori et al., 2009). In addition, individuals with AMS, and not those without AMS, had higher BP levels and BP load changes after an ascent to 3,700 m altitude (Chen et al., 2022). In this study, excessive BP load variations are associated with AMS, and BP load was suggested to be an effective indicator of systemic circulation status in AMS patients. On the other hand, it should be noted that chronic and intermittent exposure to hypoxia does not induce an increase in BP (in healthy miners) (Vinnikov et al., 2016b).

Changes in vascular endothelial cell function may also act as an important early warning sign and diagnostic index and play an important role in the occurrence and development of AMS. In the Boos et al. (2016a) study, ET-1 and SpO_2_ were demonstrated independent predictors of both AMS and its severity, however, the overall risk prediction was relatively weak.

Finally, no significant relationship was found between changes in CBF (or velocity) and AMS, and the question could still be asked (Ainslie et al., 2008; Smirl et al., 2014; Subudhi et al., 2014). Indeed, an increased CBF may contribute to the development of AMS in the face of other risk factors (e.g., metabolic/genetic profile, sleep, exercise) (Ainslie and Subudhi, 2014).

### 3.2 Cellular, inflammatory and metabolic responses

Inflammation plays a key role in the physiological response to several hypoxic stress conditions including high altitude exposure. At the cellular level, hypoxia can induce the expression of several inflammatory mediators that signal tissue damage and initiate survival responses (Eltzschig and Carmeliet, 2011). However, while hypoxia-induced inflammation can play a protective role by initiating an immune response and promoting tissue healing, it can also contribute to several high-altitude pathologies, particularly in the context of chronic hypoxia (Pham et al., 2021). Several studies have shown increases of pro-inflammatory cytokines (TNF-α, IL-1β, and IL-6) plasma levels or mRNA expression in humans exposed to high altitude (3,860 m) (Song et al., 2016; Lundeberg et al., 2018; Kammerer et al., 2020; Pham et al., 2021).

The main regulators of the transcriptional response to hypoxia and inflammation involve the HIF and NF-κB pathway, and adenosine may play a role at the interface (Eltzschig and Carmeliet, 2011; Bartels et al., 2013; Bowser et al., 2017). Cellular adaptations to hypoxia rely on HIF, which is inactive when oxygen is abundant but is activated in hypoxic conditions (Eltzschig and Carmeliet, 2011). HIF is a heterodimeric complex consisting of one of the oxygen-regulated α-subunit isoforms (HIF-1α, HIF-2α or HIF-3α) and the constitutively expressed subunit HIF-1β (Semenza, 2010). HIF-1 controls acute adaptation to hypoxia, while HIF-2 and HIF-3 activity begins later, during prolonged hypoxia, allowing transient switching between HIF proteins (Koh and Powis, 2012). When HIF-1 levels decrease, those of HIF-2 and HIF-3 increase, and the shift from HIF-1 signaling to that of HIF-2 and HIF-3 is important to enable adaptation of the endothelium to prolonged hypoxia. Under normoxia, *de novo* synthesized cytoplasmic HIF-α is regulated by hydroxylation performed by prolyl hydroxylases (PHDs) (Dzhalilova and Makarova, 2020). In hypoxic conditions, HIF-α and HIF-β subunits translocate to the nucleus, where they bind as heterodimers to a hypoxia response promoter element (HRE), inducing transcription of numerous genes, including those of NF-κB and toll-like receptors (TLRs) (Bartels et al., 2013). Still in hypoxia, the activity of HIF-degrading PHDs drops, the rate of degradation of inhibitor-kappa B (IKK) of kinase I-kappa B (IκB) increases, releasing the repression of NF-κB and allowing it to translocate to the nucleus at higher rates and to upregulate the expression of inflammatory genes as well as the HIF protein (Eltzschig and Carmeliet, 2011). There are significant differences between individuals in the degree to which expression of various HIF-regulated genes was induced by hypoxia, with an induction pattern common to all genes (Brooks et al., 2009). Individual variability in the expression of HIF and its dependent genes in response to hypoxic or inflammatory stimuli is, at least in part, due to genetic polymorphisms (van Patot and Gassmann, 2011; Zhang et al., 2014). It has been recently demonstrated in healthy participants, that an subacute (≤24 h) and prolonged (72 h) exposure to sedentary hypobaric hypoxia (direct ascent to 3,830 m) transiently increased HIF-1α mRNA levels peaking after 24 h, whereas HIF-2α mRNA levels increase after 72 h, and the NF-κB mRNA levels remained unchanged (Malacrida et al., 2019). In this study, only the circulating protein concentration of IL-1β increases slightly and transiently after 24 h of exposure to hypobaric hypoxia.

The HIF pathway is also involved in cellular metabolism and contributes to the suppression of oxidative metabolism (Murray et al., 2018). In high altitude populations, fatty acid oxidation is abandoned in favor of a metabolism adapted to the lower oxygen availability. Genetic variants underlie these adaptations (Horscroft et al., 2017; Murray et al., 2018).

In recent decades, several studies have provided evidence that hypoxia/HIF signaling is closely related to adenosine signaling (in acute and chronic lung injury, for example) (Eltzschig and Carmeliet, 2011). At the cellular level, adenosine can be transported into the cell by equilibrative nucleoside transports (ENTs), and adenosine activates adenosine receptors (A_1_R, A_2A_R, A_2B_R, A_3A_R) playing a crucial role in different cells and organs. Under normoxic conditions, adenosine has a high affinity for adenosine receptors and ENTs. Under hypoxia, the release of extracellular ATP/adenosine diphosphate (ADP) increases, HIFs promote extracellular adenosine accumulation through inhibition of ENTs, and HIFs activate adenosine receptors, thereby modulating tissue barriers and inflammatory response (Li et al., 2020). Animal and human studies have shown that adenosine levels increase after exposure to hypoxia (Liu et al., 2016; Song et al., 2017). In Drumm et al. (2004) demonstrated in humans that plasma adenosine concentrations during an hypoxic condition are significantly correlated with HVR, indicating a possible role of endogenous adenosine in the regulation of breathing.

#### 3.2.1 Cellular, inflammatory and metabolic responses and AMS

The higher levels of inflammatory biomarkers and the one of the circulating epinephrine were found associated with symptoms of AMS (Kamimori et al., 2009; Lundeberg et al., 2018; Wang et al., 2018; Kammerer et al., 2020). Moreover, it has been shown that acting pharmacologically on adenosine, a catabolite of ATP, improves the AMS: theophylline, an adenosine A_1_ and A_2_ receptor inhibitor, was found to improve AMS during exposure to chamber decompression to a simulated altitude of 4,500 m (Fischer et al., 2000). However, the exact biological mechanism involved in AMS remains to be elucidated.

The susceptibility to AMS is related to genetic background. Comparison of symptom scores and physiological parameters between subjects (healthy young non-Tibetan Chinese men residing in the plains) at 500 m and 3,700 m (airplane ascent) showed that the rs4953348 polymorphism of the *EPAS1* gene (encoding HIF-2α) significantly correlated with SaO_2_ level and AMS (Yu et al., 2016). Several studies have also shown associations of single nucleotide polymorphisms in the *NOS3* gene [encoding the nitric oxide synthase 3 which produces nitric oxide (NO) and is involved in vascular smooth muscle relaxation] with AMS susceptibility (Wang et al., 2010; Hannemann et al., 2021).

### 3.3 Cognitive responses

Hypoxic conditions at high altitude can have adverse effects on the cognitive performance of human subjects (Virués-Ortega et al., 2004; Yan, 2014; McMorris et al., 2017). In the 1930s, the first observation of cognitive disorders under hypoxia was reported by studying reaction times when performing cognitive tasks (Fleming, 1933). Many studies concerning the effects of high altitude on cognitive functions have been published in different populations such as climbers, trekkers, military personnel, flight crews and high-altitude residents (Abraini et al., 1998; Shukitt-Hale et al., 1998; Hill et al., 2014; Nation et al., 2017; Shaw et al., 2021). Although there are differences in the types of hypoxia exposure, duration, modality, and severity, neurocognitive impairments are consistently reported. Hypoxia at medium and high altitudes results in cognitive and psychomotor deficits (e.g., learning, reaction time, decision making, and certain types of memory) (Virués-Ortega et al., 2004; Wilson et al., 2009; Petrassi et al., 2012; Yan, 2014; McMorris et al., 2017).

The cognitive performance under acute exposure to altitude has been investigated on subjects during high altitude expedition or during simulated altitude chambers in laboratory. In Kramer et al. (1993) evidenced that the climbers’ rate of learning in new skills was impaired relative to that of the control subjects during and after ascent to 6,194 m of altitude. The deficits occurred in perceptual as well as semantic memory tasks. In a recent study, in 2015, Davranche et al. (2016) reported deleterious consequences on information processing in terms of speed and accuracy during 4 days exposure to 4,350 m of altitude. Slower reaction time occurs in the early hours of exposure, which is no longer detectable after 2 days, while decision errors were higher than at sea level during the entire sojourn. The acute exposure to high altitude in hypoxic chambers also negatively impacts cognitive capacities. In military personnel, mood and cognitive/ motor performance are affected during a 4.5 h exposure to three different levels of hypobaric hypoxia (500 m, 4,200 m, and 4,700 m), and the severity of the effects increases significantly at 4,700 m compared to 4,200 m (Shukitt-Hale et al., 1998). In this study, the administered tasks required cognitive processes inherent in many real-world tasks such as maintaining sustained attention, applying prior knowledge to problems, processing spatial and verbal information, performing mathematical calculations and decision making. Further, among specific cognitive task components, mathematical calculation and auditory monitoring appeared particularly vulnerable in military personnel exposed to acute moderate (5,486 m) and severe (7,620 m) hypobaric hypoxia (Beer et al., 2017). During and at the cessation of a brief (15 min) exposure to a high-altitude (6,096 m) simulation in a hypobaric chamber, a predominant impairment of learning and memory occurs, associated with slight distortions of visual and perceptual organization, and a relative preservation of basic visual and auditory attention (Nation et al., 2017). In addition, participants failed to form and consolidate (i.e., encode) new information into memory. Recently in Pramsohler et al. (2017) evidenced that the cognitive part of reaction time was impaired after sleeping one night at simulated 3,500 m and 5,500 m in comparison with sea level, while no changes were detected in the motoric part of reaction time. Finally, the systematic review and meta-regression analysis of McMorris et al. (2017) showed that acute hypoxia negatively affect cognition, whether it involves central or non-executive perception/attention or short-term memory. Low PaO_2_ (35–60 mmHg) was the main predictor of cognitive performance, regardless of whether exposure occurred under hypobaric or normobaric hypoxic conditions.

In summary, the cognitive impairments associated with acute and gradual altitude exposure were schematized in the Wilson et al. (2009) review and remain relevant: decreased complex reaction time at medium altitudes up to 2,000–3,000 m, impairments in psychomotor abilities at 3,000–4,000 m, impairments in working memory and learning at 4,000–6,000 m, and hallucinations at extreme altitudes above 7,500 m. The study by Kryskow et al. (2013) illustrated one of the above points by showing that simple psychomotor performance in the military task of disassembling and reassembling a weapon was unaffected between 2,500 and 4,300 m, whereas complex psychomotor performance (i.e., rate of fire with a rifle) was degraded at 4,300 m, and this was most likely due to an increase in sleepiness (Kryskow et al., 2013).

In the central nervous system, neurons are very sensitive to the availability of oxygen (Bailey et al., 2017), and failure of synaptic transmission can be observed within minutes of acute hypoxia (Corcoran and O’Connor, 2013). The acute effects of hypoxia on synaptic transmission are mediated primarily by alterations in ion fluxes across membranes, presynaptic effects of adenosine and other actions at glutamatergic receptors (Corcoran and O’Connor, 2013). Several mechanisms are involved in the cognitive impairment after both acute and chronic hypoxia, such as glycolysis, the rapid increase of brain adenosine and related activation of receptors, oxidative stress, calcium overload and inflammation, but substantial differences exist in response to these in two hypoxia conditions (Massaad and Klann, 2011; Chen et al., 2018; Wang et al., 2022). With respect to adenosine receptors, genetic inactivation of A_2A_ receptors in a model of mice prevented spatial memory impairment in an acute hypobaric hypoxia (7 days to 8,000 m) (Chen et al., 2018). During chronic hypoxia, the mechanisms of oxidative stress and inflammation are somewhat different from those of acute hypoxia, and some signaling pathways mediated by transcription factors are involved (Wang et al., 2022). In addition, there are also mechanisms acting specifically in the chronic stage of hypoxia, with adaptive responses. These involve an increase in Hb concentration and hematocrit (after upregulation of erythropoietin), angiogenesis, and adaptive mitochondrial responses.

Finally, the brain is a high-flow organ that depends on acute increases in regional blood flow and oxygen supply to sustain increases in neuronal activity, a process known as neurovascular coupling. Partial pressure of carbon dioxide in arterial blood and/or acid-base status (i.e., alkalosis) was found to influence not only cerebrovascular hemodynamics but also cognitive performance (Friend et al., 2019; Leacy et al., 2019).

## 4 Physiological and cognitive responses to hypoxic hypoxia after sleep loss

**FIGURE 1:**
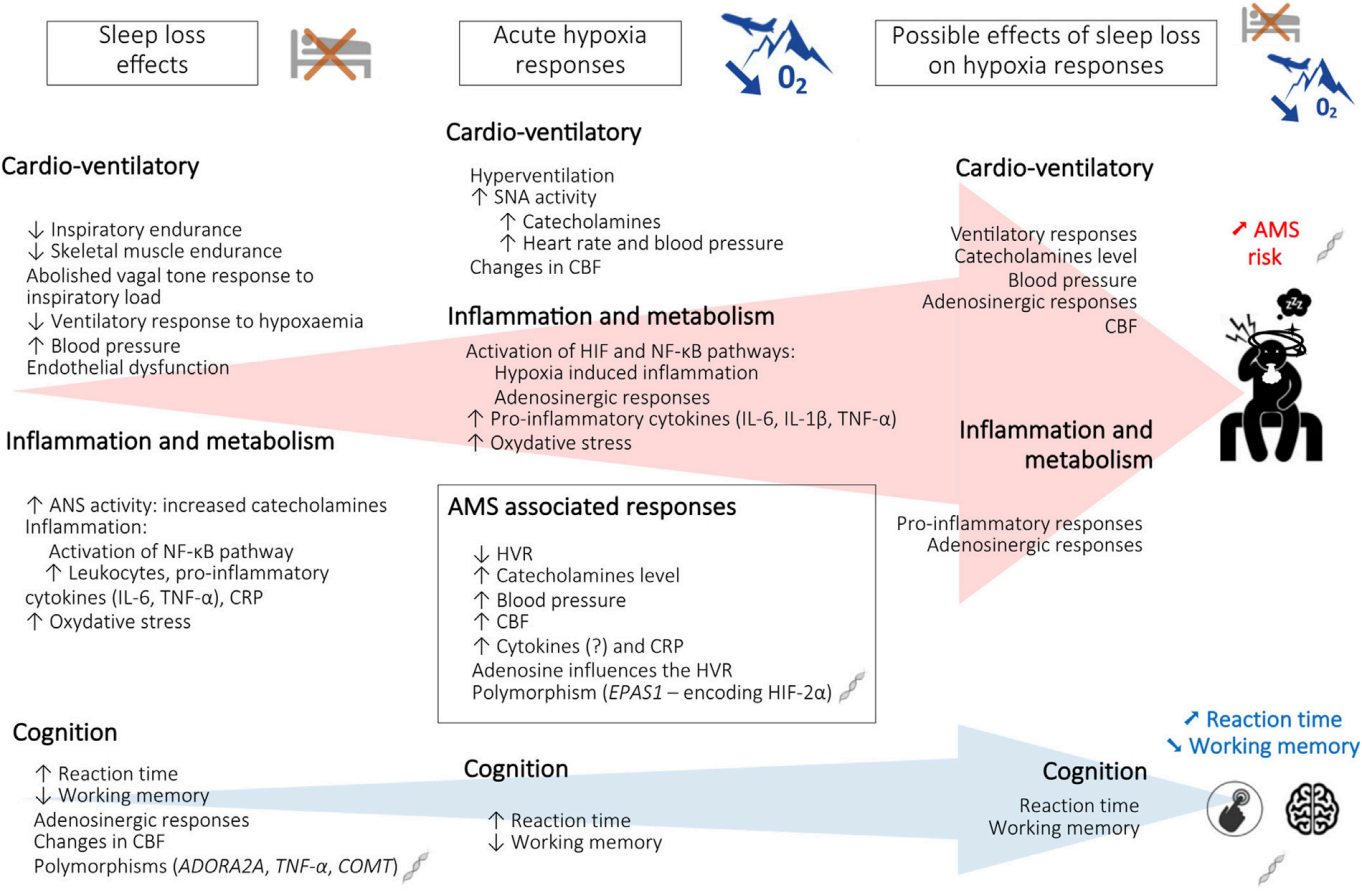
Schematic representation of the effects of sleep loss and hypoxia on altitude tolerance [acute mountain sickness (AMS) and cognition]. *ADORA2A*, Adenosine A_2A_ receptor gene; ANS, autonomic nervous system; CBF, cerebral blood flow; *COMT*, Catechol-O-methyltransferase gene; CRP, C-reactive protein; *EPAS1*, Endothelial PAS domain-containing protein 1 gene; HIF-2α, hypoxia-inducible factor-2α; HVR, hypoxic ventilatory response; IL-6, interleukin-6; IL-1β, interleukin-1β; NF- κB, Nuclear factor-kappa B; O_2_, oxygen; TNF-α, tumor necrosis factor alpha; *TNF-α*, Tumor necrosis factor alpha gene.

Data from the first two chapters of this review show that SD and exposure to environmental hypoxia influence respiratory/cardiovascular and metabolic/inflammatory/hormonal responses (Spengler and Shea, 2000; Mullington et al., 2009; Chennaoui et al., 2020). These different points of interference between SD and responses to hypoxia make it possible to formulate hypotheses on the alteration of physiological tolerance, the risk of AMS, and the deterioration of cognitive performance, when these two constraints are combined (Figure 1). In this sense, Figueiredo et al. (2022) demonstrated that AMS symptoms have a greater negative impact on mood while poor sleep has a greater negative impact on cognition, in unacclimatized lowlanders exposed to either 3,000 m or 4,050 m for 20 h in a hypobaric chamber.

### 4.1 Respiratory responses and AMS

In the recent study by Rault et al. (2020) and Anderson et al. (1995), progressive reduction of cortical contribution to respiratory motor output was associated with SD-related decrease in inspiratory endurance, with unaffected diaphragmatic contractile function. Thus, SD could have a negative impact on physiological responses to environmental hypoxia, e.g., desensitization of the physiological ventilatory response. In 2007, it was shown that respiratory muscle strength (assessed by maximal inspiratory pressure) is transiently reduced during the 4-day progressive ascent to 4,559 m in the Italian Alps, with values always remaining within an acceptable range to avoid true muscle fatigue, and without reports of severe AMS (Fasano et al., 2007).

As the ventilatory response to hypoxia appears to be linked to the adenosinergic system, it is possible that this system, or even its genetic determinism, has an impact on ventilatory adaptations during the combination of stressors, SD *plus* hypoxia exposure. We therefore postulated that lack of sleep prior to ascent would have an additional deleterious effect on the acute and sustained ventilatory response to hypoxia and could increase the risk of AMS.

### 4.2 Cardiovascular responses and AMS

Some studies have shown that insufficient sleep can disturb cardiovascular responses to environmental hypoxia in healthy humans. In the animal model, subchronic and chronic exposure to intermittent hypoxia combined to SD was found to modify biochemical blood parameters relative to cardiovascular risk in distinct ways (Perry et al., 2007). Moreover, a recent study demonstrated that the combined exposure to a rotational work with a chronic intermittent hypoxia due to altitude (e.g., for mining workers) (between 2,600 and 3,600 m above sea level and off duty days at <800 m) induced a higher cardiovascular risk compared with workers exposed to rotational work shift alone (Pizarro-Montaner et al., 2020). Finally, a potentiation of the increase of heart rate induced by SD and by an inspiratory loading task as observed in hypoxia, which may be due to a reduced adaptive response of vagal tone (Westphal et al., 2021).

These observations suggest that SD associated with exposure to hypoxia can increase cardiovascular risk and the occurrence of cardiovascular events, already favored by the decrease in tissue PO_2_. We also hypothesize that the increase in blood pressure following SD would favor the occurrence of AMS, this pathology being associated with higher blood pressure values.

### 4.3 Inflammatory and metabolic responses and AMS

SD is associated with an increase in the level pro-inflammatory markers in blood (CRP but also cytokines, chemokines) and tissues (e.g., in the brain). SD also reduces the Th1 response and increases the Th2 response, alters the presentation of the antigen by the reduction of dendritic cells and reduces the production of antibodies (Besedovsky et al., 2012; Irwin, 2019). The sympathetic nervous system (SNS) and HPA are main effector systems that link sleep disturbances and immunity. DS is associated with SNS activation and systemic catecholamine release, but to some extent HPA stimulation may be impaired. Alterations in SNS and HPA associated with reactive oxygen species and adenosine trigger inflammatory activation of brain and tissue immune cells through the activation of transcriptional regulators of pro-inflammatory gene expression (primarily NF-κB) (Irwin, 2019). Yet, hypoxia is also known to induce inflammatory responses and cytokine release (Lundeberg et al., 2018; Wang et al., 2018; Kammerer et al., 2020). Furthermore, there is significant crosstalk between the transcription factors NF-κB and HIF involved in inflammation and hypoxia (Bandarra et al., 2015). Therefore, we can hypothesize that a prior pro-inflammatory state (even low-grade) due to SD will be detrimental upon exposure to high altitude with the risk of developing AMS, itself associated with inflammation (Wang et al., 2018).

### 4.4 Cognitive responses

Cognitive impairments, particularly increased reaction time, may be induced by both SD and exposure to acute or chronic hypoxia, and potentially exacerbated or recovery delayed if these stresses are successively accumulated, sharing possibly common mechanisms including brain adenosine, inflammation, oxidative stress, and activation of the HIF and NF-κB pathway. Indeed, in 1986, it was described that there was a significant interaction between SD and altitude (simulated by gas mixtures, 3,810 m) that was enhanced by increasing workload in a complex performance task (Mertens and Collins, 1986). When the subjects were sleep deprived (one night), performance was significantly lower, and the greatest decrement in performance occurred at altitude. It therefore seems relevant to limit sleep deprivation before exposure to environmental hypoxia, or even to create a sleep capital for oneself. Our previous results showed that “banking” about 1 h of sleep during the seven nights prior to total sleep deprivation limits the degradation of sustained attention in healthy subjects, and the “banking sleep” concept emerged (Arnal et al., 2015; Axelsson and Vyazovskiy, 2015; Yarnell and Deuster, 2016; Walsh et al., 2020). It has even been shown that “banking sleep” is beneficial for motivation during cognitive tasks and fatigue in healthy young adults with normal sleep patterns (Ritland et al., 2019; Mantua et al., 2021), and that it is feasible in healthy, free-living young adults, who are habitually short-sleepers, underlying the utility of including sleep hygiene guidelines in public health messages (Al Khatib et al., 2018). The influence of other behavioral countermeasures used to prevent the deleterious effects of SD (napping, blue-enriched light, caffeine consumption) on cognition (Arnal et al., 2015; Faraut et al., 2017; Faraut et al., 2020; Erblang et al., 2021a) would merit investigation with the goal of mitigating the potential exacerbation induced by the combination of SD and hypoxia stressors. The very first recommendation for people who will be exposed to hypoxia (professionally or recreationally) would be not to be in SD before the exposure, at least with respect to cognitive performance.

In addition, in order to propose personalized countermeasures, differences in responses associated with genetic polymorphisms should also be taken into account. Several genetic polymorphisms related to adenosine receptors (*ADORA2A*), inflammatory biomarker (*TNF-α*), or catecholamine functioning (*COMT*), have been associated with neurobehavioral resilience and vulnerability to SD (Casale and Goel, 2021), and other polymorphisms related to hypoxia tolerance such as *HMOX2* (implicating possible adaptive regulation of Hb levels at high altitude), and hypoxia-induced AMS such as mutations in HIF 2 (*EPAS1*) (Yang et al., 2016; Yu et al., 2016; Fabries et al., 2022). It will thus be interesting to further investigate genetic influences on the combined stressors of SD and hypoxia.

### 4.5 Practical recommendations for operational military personnel

Military personnel in particular (mountain combat forces, security and emergency services, helicopter crews, pilots) may be acutely exposed to altitudes at the threshold for acute hypoxia disorders (3,500 m), being sleep deprived, sometimes even associated with additional constraints (climatic, nutritional, psychological, physical and mental). This can have deleterious consequences on their cognitive and psychomotor operational performances, alone or in groups, with a one-off risk of AMS, but also a medium and long-term risk of metabolic and cardiovascular diseases in the event of repeated missions without adequate recovery.

Awareness of the multiple consequences (physiological and operational) of sleep deprivation and hypoxia in military personnel has become essential prior to missions at altitudes in the range of 3,000–4,000 m, knowing that the risk of AMS may be present. The development of sleep interventions, such as sleep extension, is important to limit cognitive impairment and hypoxia-related fatigue, and improve motivation.

With respect to high altitude-related AMS risk, gradual ascent is the cornerstone of prevention, and current recommendations are to avoid abrupt ascents to altitudes higher than 3,000 m (sudden increases of greater than 600 m should be avoided) and to spend 2 or 3 nights at 2,500–3,000 m before further ascent. In addition, the physical exhaustion had to be avoided and a high-carbohydrate diet may benefit to the metabolic demand of exercise at high altitude, and may aid in acclimatization (Klocke et al., 1998; Hill et al., 2011; Luks et al., 2017). In the aeronautical context, to improve flight safety, pilots and crewmembers flying on unpressurized aircraft could receive oxygen supplementation from the first 30 min at pressure altitudes above 3,048 m [COMMISSION REGULATION (EU), 2013; Code of Federal Regulation, 2022; NATO, 2017], in particular when personnel are exposed to a combination of operational constraints (night flights, training periods for parachutists, fatigue, etc.) or during repeated exposures. Finally, all of these recommendations should be tailored to individual intrinsic factors.

## 5 Conclusion

To our knowledge, this review is the first to present data in healthy humans regarding the effects of sleep loss and exposure to environmental hypoxia on their most common physiological and cognitive responses in relation to the risk of AMS, and to hypothesize a potentiation of these effects in a sleep-deprived individual during high altitude exposure. In this regard, a recent study evaluating the latest version of the Lake Louise AMS score in a cohort of 484 high altitude hikers emphasizes the item “sleep restriction” as a contributor to AMS diagnosis (Richalet et al., 2021). Therefore, our future laboratory protocols should assess the impact of sleep deprivation/restriction on physiological and cognitive responses (i.e., those defined in this review) to prolonged acute normobaric hypoxia, using counterbalanced cross-over designs. There is also a need to investigate ecologically valid protocols for the sleep deprivation condition. This research would allow us to formulate non-pharmacological advice primarily on sleep management before exposure to high altitude, towards professionals or even for leisure activities. A final point that should be explored is the interaction between sleep deficit and additional constraints (e.g., physical and psychological load, nutrition/hydration, thermal conditions) on physiological and cognitive responses to environmental hypoxia.
